# BMC Urology reviewer acknowledgement, 2013

**DOI:** 10.1186/1471-2490-14-15

**Published:** 2014-02-27

**Authors:** Hayley Henderson

**Affiliations:** 1 BioMed Central, Floor 6, 236 Gray’s Inn Road, London WC1X 8HB, United Kingdom

## Abstract

**Contributing reviewers:**

The editors of BMC Urology would like to thank all our reviewers who have contributed to the journal in Volume 13 (2013).

## 

A Kahokehr

Aruba

Adam Metwalli

USA

Adam Luchey

USA

Adam Reese

USA

Adrian Fairey

Canada

Ahmed Ghaith

Egypt

Ahmed El-Zawahry

USA

Ahmet Yaser Muslumanoglu

Turkey

Akihiro Kanematsu

Japan

Akira Miyajima

Japan

Aleksander Giwercman

Sweden

Alessandro Anselmo

Italy

Alessandro Calarco

Italy

Alexander Parker

USA

Allen Gao

USA

Alvis Laukmanis

Latvia

Amanda Sacker

UK

Andrea Minervini

Italy

Andreas Røder

Denmark

Andrew Armstrong

USA

Angelo Territo

Italy

Antonio Cicione

Italy

Asaf Biber

Israel

Ashraf Hassan

Egypt

Asli Serter

Turkey

Badar Mian

Italy

Bahareh Vahabi

UK

Baris Turkbey

USA

Barry Stein

USA

Bidya Shrestha

Nepal

Bimal Bhindi

Canada

Binay Shah

USA

Brian Flynn

USA

Brian Chapin

USA

Carlos Estrada

USA

Cecilia Maria Cracco

Italy

Cesur Gumus

Turkey

Charles Rosser

USA

Chris Protzel

Germany

Christos Lionis

Greece

Claudia Calì

Italy

Costantino Leonardo

Italy

Daniel Moreira

USA

Daniel Su

USA

Daniel Canter

USA

Daniel Fernandez

USA

Danny Youlden

Australia

David Thiel

USA

David Edwards

UK

David Ralph

UK

David Degraff

USA

Debra E. Irwin

USA

Deepika Dhawan

USA

Dermot Leahy

Ireland

Diana Marshall Phd

UK

Dogu Teber

Germany

Donald Lamm

USA

Elizabeth Casiano

USA

Emily Qualls-Creekmore

USA

Enrico Finazzi Agro

Italy

Eugene Pietzak

USA

Eugenio Martorana

Italy

Fabrizio Presicce

Italy

Fadi Brimo

Canada

Ferdinand Frauscher

Austria

Francesco Berardinelli

Italy

Francesco Celestino

Italy

Francesco Bottone

Italy

Francis Chinegwundoh

UK

Frederic Pouliot

Canada

Gaetano Ciancio

USA

Gennady Bratslavsky

USA

Geoffrey Gotto

Canada

George Alangaden

USA

Georgi Guruli

USA

Gianmarco Isgro

Italy

Giorgio Ivan Russo

Italy

Giovanni Battista Di Pierro

Italy

Giuseppe Procopio

Italy

Gou Kaneko

Japan

Guillaume Ploussard

France

Hans Heinzer

Germany

Heinric Williams

USA

Hendrik Isbarn

Germany

Hiroshi Miyamoto

USA

Hitoshi Masuda

Japan

Isabel Pascual

Spain

J Coleman

USA

Jae Hyun Bae

Korea, South

Jae Y. Ro

USA

Jae Young Park

Korea, South

James Malone Lee

UK

Jan Wohlfahrt

Denmark

Jehonathan Pinthus

Canada

Jian Gong

China

Joanne Booth

UK

John Lavelle

USA

John F Mcdonald

USA

Jonas Worsoe

Denmark

Jonathan Izawa

UK

Joshua Meeks

USA

Junfeng Wang

USA

Justin Ziemba

USA

Kazim Serhan Ozcan

Turkey

Kenneth Iczkowski

USA

Kivanç Bektas Kayhan

Turkey

Krishnan Venkatesan

USA

Lalgudi Narayanan Dorairajan

India

Lambros Stamatakis

USA

Long-Cheng Li

USA

Luca Cindolo

Italy

Luigi Cormio

Italy

Mahzuz Karim

UK

Majid Mirzazadeh

USA

Manuela Ingrosso

Italy

Marcello Siniscalchi

Italy

Marco Grande

Italy

Marco Rosa

Italy

Marco De Sio

Italy

Maria Morales

Spain

Maria Angela Cerruto

Italy

Marije Bomers

Netherlands

Matteo Vittori

Italy

Maximilian Burger

Germany

Mayank Agarwal

India

Mayer Fishman

USA

Mehmet Ruhi Onur

Turkey

Mi Mi Oh

Korea, South

Michael Poch

USA

Michael Fleisher

USA

Michael R Ruggieri, Sr.

USA

Michele Marchioni

Italy

Mikkel Fode

Denmark

Mindaugas Jievaltas

Lithuania

Minoru Miyazato

Japan

Mohammad Katouli

Australia

Mohummad Siddiqui

United States Minor Outlying Islands

Molly Williams

USA

Mostafa Taymour

Egypt

Nadeem Dhanani

USA

Norifumi Sawada

USA

Octavio Castillo

Chile

Onkar Singh

India

Oriol De Solà-Morales

Spain

Paul Crispen

USA

Pedro Fernandez

South Africa

Petros Sountoulides

Greece

Philippe Spiess

USA

Phillip Pierorazio

USA

Phillip Mucksavage

USA

Piergustavo De Francesco

Italy

PietroCastellan

Italy

Piyush Agarwal

USA

Rahmi Onur

Turkey

Ravimohan Mavuduru

India

Rebecca O'Malley

USA

Riccardo Galli

Italy

Riccardo Bartoletti

Italy

Richard Santucci

USA

Richard Zigeuner

Austria

Rick Kittles

USA

Roberto Castellucci

Italy

Roberto Molina

Spain

Roberto Miano

Italy

Rollin Laetitia

France

Rui Henrique

Portugal

Sae-Ock Oh

Korea, South

Saleh Binsaleh

Saudi Arabia

Salvatore Micali

Italy

Sandip Prasad

USA

Sandrine Leroy

France

Sanjay Sinha

India

Satoshi Anai

Japan

Scott Delacroix, Jr.

USA

Serkan Altinova

Turkey

Shawky Elhaggar

Egypt

Shilpa Gupta

USA

Silvia Proietti

Italy

Sima Porten

USA

Simpa Salami

USA

Souhil Lebdai

France

Stanley Yap

USA

Stefano Picozzi

Italy

Stephan Kruck

Germany

Stephen Kraus

USA

Steven Gray

Ireland

Steven Nordeen

USA

Sung Kyu Hong

Korea, South

Suresh Bhagat

India

Takeshi Inoue

Japan

Tatsuya Mimura

Japan

Thomas Vates

USA

Thomas Schwaab

USA

Thomas Chi

USA

Tomas Buchler

Czech Republic

Valéria Carvalho

Brazil

Vivek Kute

India

Wade Sexton

USA

Wasia Rizwani

USA

Wassim Kassouf

Canada

Yasushi Nakai

Japan

Yasushi Yoshino

Japan

Yeonghau Lien

USA

Yong-Rui Bai

China

Yoshihiro Tatsumi

Japan

Zachary Smith

USA

Zongbing You

USA

## Pre-publication history

The pre-publication history for this paper can be accessed here:

http://www.biomedcentral.com/1471-2490/14/15/prepub

